# Fruit flavors in electronic cigarettes (ECIGs) are associated with nocturnal dry cough: A population longitudinal analysis

**DOI:** 10.1371/journal.pone.0306467

**Published:** 2024-06-28

**Authors:** Zahira Quinones Tavarez, Daniel P. Croft, Dongmei Li, Steven R. Gill, Andrew P. Wojtovich, Irfan Rahman, Deborah J. Ossip

**Affiliations:** 1 Clinical Translational Science Institute, University of Rochester Medical Center, Rochester, New York, United States of America; 2 Department of Public Health Sciences, University of Rochester School of Medicine and Dentistry, Rochester, New York, United States of America; 3 Department of Medicine, Pulmonary Diseases and Critical Care, University of Rochester Medical Center, Rochester, New York, United States of America; 4 Department of Microbiology and Immunology, University of Rochester School of Medicine and Dentistry, Rochester, New York, United States of America; 5 Department of Anesthesiology and Perioperative Medicine, University of Rochester Medical Center, Rochester, New York, United States of America; 6 Department of Environmental Medicine, University of Rochester School of Medicine and Dentistry, Rochester, New York, United States of America; Xiamen University - Malaysia Campus: Xiamen University - Malaysia, MALAYSIA

## Abstract

Evidence from *in vitro* and animal models has identified the pulmonary toxicity of flavors in electronic cigarettes (ECIGs); however, less is known from epidemiological studies about the effects of flavors in the respiratory health. This study examined the longitudinal association between exposure to ECIGs flavors and nocturnal dry cough among ECIGs users. A secondary analysis of data from the Population Assessment of Tobacco and Health Study (2014–2019) was conducted. The study population included adults who provided information (n = 18,925) for a total of 38,638 observations. Weighted-incidence estimates and weighted- generalized estimating equation models were performed to assess unadjusted and adjusted associations. The weighted incidence proportion (WIP) of nocturnal dry cough was significantly higher among current (WIP:16.6%; 95%CI 10.5, 21.2) and former fruit flavored ECIGs users (WIP:16.6%; 95%CI 11.3, 21.9) as compared to non-ECIGs users (WIP:11.1%; 95%CI 10.6, 11.6). Current ECIGs users of fruit flavors showed 40% higher risk of reporting cough than non-ECIGs users (aRR:1.40, 95%CI 1.01, 1.94). Former ECIGs users of multiple flavors and other flavors had 300% and 66% higher risk to develop cough, respectively (aRR:3.33, 95%CI 1.51, 7.34 and aRR:1.66, 95%CI 1.0.9, 2.51), relative to non-ECIGs users. We observed a significantly higher risk of developing nocturnal dry cough in the past 12 months in current and former ECIGs users of fruit flavors and in former ECIGs users of multiple flavors. To the extent that cough may serve as an early indicator of respiratory inflammation and potential disease risk, the association between ECIGs use and cough raises potential concerns.

## Introduction

The use of electronic cigarettes (ECIGs) among youth and young adults is now a serious public health concern [1]. ECIGs are non-combustible tobacco products that heat and aerosolize a liquid containing humectants and solvents, mainly propylene glycol (PG) with or without vegetable glycerin (VG), with added flavorings and often nicotine [2, 3]. The constituents in e-liquids and the aerosols are not inert and have been reported to be associated with negative health effects. Flavorings in flavored ECIG contribute to the toxicity of e-liquids and ECIG aerosols [4–6], including a major source of toxicants such as aldehydes (formaldehyde and acetaldehyde), carbonyl emissions, reactive species of oxygen, and other free radicals [7–10]. *In vitro* and animal studies of the toxicological effects of flavored ECIG have focused on the respiratory system. Strawberry, menthol, vanillin, and cinnamon are the flavors most commonly associated with negative effects in the lungs [11]. ECIG flavorings dysregulate the respiratory innate immunity and elicit inflammatory responses, which are associated with adverse health effects in the airways. Specifically, cinnamaldehyde and menthol might disrupt the airway’s epithelia, elicit oxidative stress, impair mucociliary clearance, and induce inflammatory responses by increasing the release of inflammatory cytokines and through the activation of transient receptor potential ankyrin 1 (TRPA1) and transient receptor potential vanillin 1 (TRPV1) [11–14]. All of these pathological processes are associated with respiratory disease and symptoms, including wheezing and coughing [15, 16]. Despite assumptions that ECIGs are less harmful than combustible cigarettes, research suggests that ECIGs use has potential and actual health risks [17]. Evidence from *in vitro*, animal, and population-based studies supports the association of ECIGs use and addiction, [18, 19] cardiovascular disease, [20–23] cancer, [24–26] and respiratory disease, including acute and chronic lung damage [27–30].

The 2019 outbreak of electronic cigarettes, or vaping, product use-associated lung injury (EVALI) raised national concern in the US about the harmful effects of ECIGs products and constituents [31, 32]. As of February 2020, a total of 2807 patients were hospitalized with EVALI, and 69 deaths were reported in the United States (US) [31, 33]. Cases of EVALI in the US were linked to the presence of vitamin E acetate (VEA) in tetrahydrocannabinol containing products that were inhaled using ECIGs [31]. Though VEA is one potential cause of EVALI, more research is needed to understand the short- and long-term health effects of ECIGs constituents in e-liquids and aerosols, including the respiratory health effects [32]. There is currently a need for improved characterization of non-hospitalized ECIG users who may have subacute respiratory symptoms (e.g. mild cough or subtle dyspnea) related to their ECIGs use [32].

Monitoring respiratory symptoms among ECIGs users might help assess the potential risk of lung injury or pulmonary disease. ECIGs use among never tobacco users is associated with increased report of respiratory symptoms [34, 35]. The risk of wheezing and other respiratory symptoms has been shown to be greater among ECIGs users as compared to non-users though lower when contrasted with smokers [36]. A recent study showed that exclusive use of ECIGs was not associated with functionally important respiratory symptoms, including wheezing and coughing [37].

The National Academy of Sciences, Engineering, and Medicine reported that there is moderate evidence for increased cough and wheezing among adolescents who use ECIGs [38]. ECIGs users are more likely than non-users to report persistent cough [39] and to show inhibition of the cough reflex sensitivity [40] and transient inhibition of the urge-to-cough sensation [41, 42]. Few reports have focused on the effects of ECIGs use on coughing [40–42]. Briefly, coughing is one of the most common symptoms reported by ECIGs users. A recent report indicated a higher prevalence of respiratory symptoms among never tobacco youth and non-daily ever smokers in Canada, with 40.1% of daily ECIGs users and non-daily ever tobacco users reporting regular cough in the past 4 months [43]. Initially, concerns regarding ECIGs exposure were focused on the effects of nicotine; however, exposure to PG, VG, and flavorings from ECIGs are of special concern and merit further research to examine the harmful effects of ECIGs, including coughing. This study examined the longitudinal association between exposure to flavors in ECIGs and nocturnal dry cough among ECIGs users.

## Materials and methods

This study involved a secondary analysis of publicly available longitudinal data from the Population Assessment of Tobacco and Health (PATH) Study (2014–2019) [44]. The current study utilized publicly available and unidentifiable data which qualifies as IRB exempt review.

### Study design

The PATH Study has been described elsewhere [45]. Briefly, the PATH Study is a nationally representative, ongoing, prospective longitudinal cohort of non-institutionalized adults (aged 18 and older) and youth (aged 12–17) in the United States (US). The PATH Study uses a four-stage stratified area probability sample design to select individual participants. A total of 45,971 adults aged 18 and older were recruited in Wave 1 of the PATH Study. At the time of Wave 4, a replenishment sample was selected and combined with Wave 1 participants. Full sample weights are provided to account for the complex sample design and nonresponse. Replicate weights are also provided to improve statistical precision of the estimates. The PATH Study collects data on tobacco use and health outcomes to inform tobacco regulatory efforts. All adult participants provided informed consent. The PATH Study is conducted by Westat and ethically approved by the Westat Institutional Review Board. The current analyses include data collected from Wave (W) 1 (2013–2014), W2 (2014–2015), W3 (2015–2016), W4 (2016–2018), and W5 (2018–2019). Audio computer assisted surveys were conducted 1 year after the previous wave for all waves, except W5, which was conducted 2 years after W4. The PATH Study collects data on tobacco use and health outcomes to inform tobacco regulatory efforts.

### Study sample

**Fig 1:**
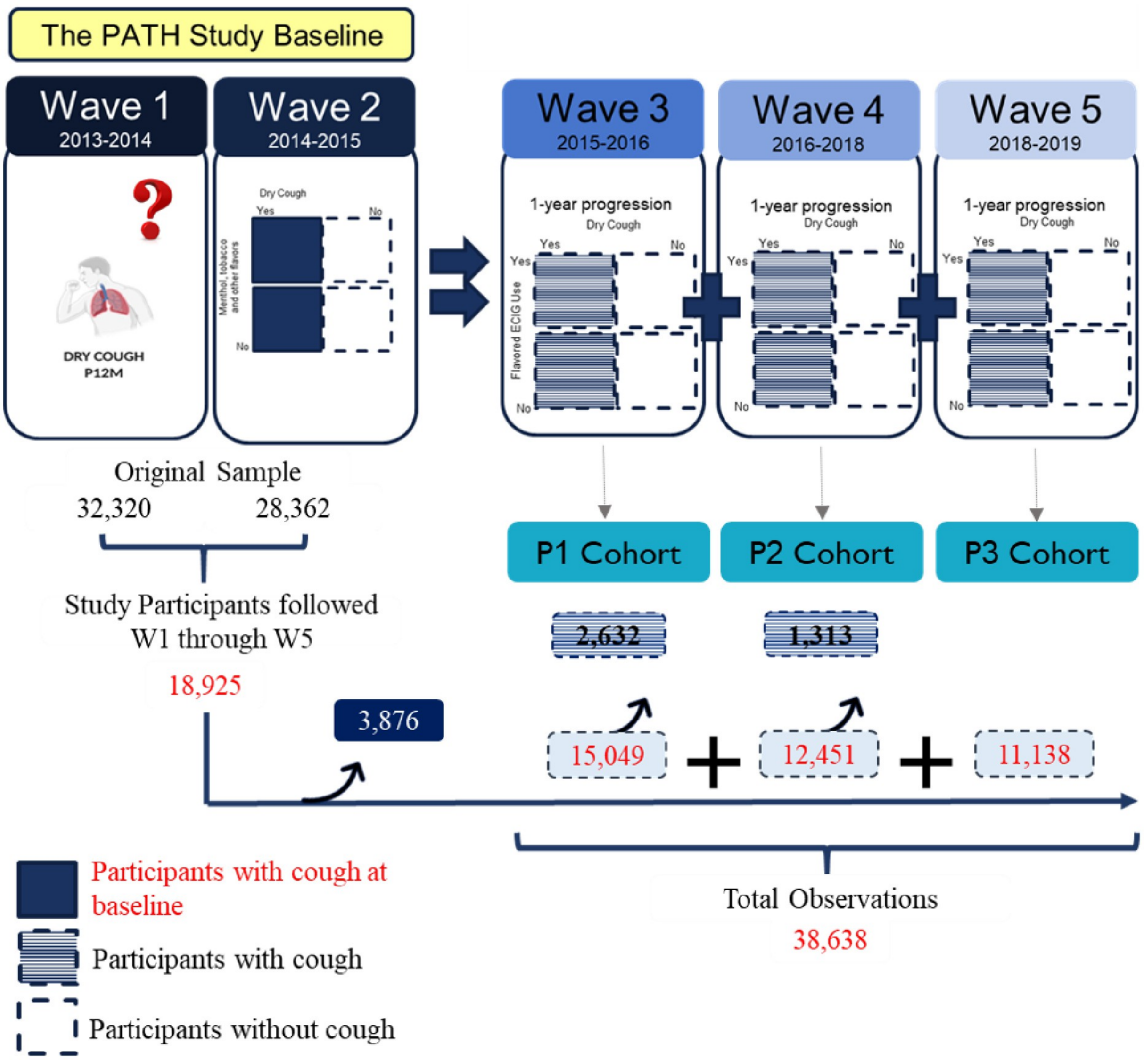
Study design and sample. The study included participants who were followed across five waves of the PATH Study and considered new cases of coughing across three period cohorts (P1-P3). Each period represents a one-year follow-up period per participant. In each period, participants with missing information about cough and cases of cough were excluded. Final sample included 18,925 participants who were followed across the five waves, who contributed to a total of 38,638 observations.

The study sample included W1 adult participants who were interviewed at all 5 waves to enable the study to generate weighted estimates based on the all-wave weights assigned to participants in W5. However, the baseline analytic sample was restricted to W2 participants due to lack of assessment of the outcome variable in W1 (n = 18,925 participants). Three 1-year interval exposure periods (P) were established for the study: P1 (W2-W3), P2 (W3-W4), and P3 (W4-W5). In each period, participants who did not report dry cough during baseline (W2 in P1, W3 in P2, and W4 in P3) were followed onto next wave to provide data equivalent to 1 observation. All observations from every participant provided a total analytical sample of 38,638 observations (Fig 1).

### Variables and measures

#### Dependent variable

The outcome variable was self-reported dry cough in the past 12 month (P12M). Nocturnal cough is a common symptom associated with environmental exposure such as tobacco smoke and secondhand smoker exposure [46]. Among the functionally important respiratory symptoms assessed by the PATH Study, only one question examines cough, specifically, nocturnal dry cough [37]. Participants who responded yes to the question “*In the past 12 months*, *have you had a dry cough at night*, *apart from a cough associated with a cold or chest infection*?” were considered as having dry cough in the P12M. A slightly different wording was used from W3 onwards: “*A dry cough is a cough without phlegm or mucus*. *In the past 12 months*, *have you had a dry cough at night*?”

#### Independent variables

Respondents reported if their regular or last brand of e-cigarette was flavored to taste like menthol, mint, clove, spice, fruit, chocolate, alcoholic drinks, candy or other sweets. Tobacco flavor was added to the list of options in W3. Six mutually exclusive categories of flavors used among current and former established e-cigarette users were created: menthol or mint flavors, tobacco, fruit, candy and sweet, multiple (two or more flavors reported), and others (clove or spice, chocolate, and alcoholic drinks flavors and other not specified in the question). Respondents were allowed to select more than one flavor. Based on the use of flavored ECIG products, participants were categorized as non-ECIG users, those who did not report established use of ECIG products, and those ECIG users reporting different flavor categories.

#### Potential confounders

The following variables were included in all multivariable analyses to account for potential confounding: sex, age, race and ethnicity, educational level, household income, body mass index (BMI), disease status, overall tobacco use, marijuana use, and secondhand smoke exposure status; these variables were statistically associated with the independent and dependent study variables. Categories for variables are provided in Table 1. Disease status included any heart condition, any lung disease, gum disease, cancer, precancerous oral lesions, ulcer, gastrointestinal bleeding, cataract and glaucoma, or diabetes mellitus in the P12M. Overall tobacco use includes four mutually exclusive categories: 1) never tobacco users (adult respondents who have never smoked or used a tobacco product); 2) ever non-established tobacco users (adult respondents who have used tobacco products but do not meet the threshold for established tobacco use and/or currently do not use the product every day or some days); 3) current established, and 4) former established tobacco users.

**Table 1:** Sociodemographic and health characteristics of participants included in the analysis, PATH study, Waves 1–5 (2013–2019), n = 18,925 individual participants and 38,638 observations.

Variables	Baseline Individual Characteristics (Wave 2)			Observations Characteristics Follow-up Period		
	Frequency ^a^	Weighted Frequency	% (95% CI)	Frequency ^b^	Weighted Frequency	% (95% CI)
Age						
18–24	4,305	25,420, 078	11.3 (11.1, 11.5)	6,041	36,908,320	7.6 (7.3,7.8)
25–34	4,110	41,129,441	18.3 (17.7, 19.0)	10,293	91,538,357	18.8 (18.0, 19.5)
35–44	3,066	38,161,051	17.0 (16.4, 17.6)	6.564	84,757,295	17.4 (16.7, 18.1)
45–54	3,024	39,522,989	17.6 (17.0, 18.3)	5,856	85,291,985	17.5 (16.7, 18.3)
55–64	2,629	39,733,956	17.7 (17.1, 18.3)	5,369	87,696,823	18.0 (17.3, 18.7)
≥65	1,788	40,150,581	17.9 (17.5, 18.4)	4,512	101,572,728	20.8 (20.1, 21.5)
Sex						
Male	8,949	107,084,660	47.8 (47.6, 48.0)	18,828	237,482,680	48.7 (48.2, 49.2)
Female	9,959	116,875,901	52.2 (52.0, 52.4)	19,775	249,847,998	51.3 (50.7, 51.7)
Highest educational level achieved						
High school or less	7,403	84,252,983	37.7 (37.2, 38.3)	13,266	166,913,832	34.3 (33.5, 35.1)
Some college or associate degrees	6,929	73,519,156	32.9 (32.2, 33.6)	13,349	151,124,568	31.1 (30.4, 31.8)
Bachelor’s degrees or higher	4,522	65,601,815	29.4 (29.0, 29.8)	11,910	168,186,631	34.6 (33.9, 35.3)
Currently covered by health insurance						
Yes	15,828	196,190,621	87.8 (87.2, 88.4)	29,813	399,856,608	82.6 (81.9, 83.2)
No	1,416	27,192,699	12.2 (11.6, 12.8)	4,205	44,903,040	9.3 (8.8, 9.8)
Total household income in the past 12 months (US$)						
<10,000	3,133	25,171,651	12.1 (11.5, 12.8)	4,573	39,296,857	8.7 (8.2, 9.2)
10,000–24,999	4,006	40,804,595	19.7 (18.8, 20.6)	6,840	74,416,767	16.4 (15.8, 17.1)
25,000–49,999	4,090	47,364,471	22.9 (21.9, 23.8)	8,584	99,292,710	21.9 (21.1, 22.8)
50,000–99,999	3,953	54,503,448	26.3 (25.3, 27.2)	9,670	132,356,493	29.2 (28.3, 30.2)
>100,000	2,572	39,311,517	19.0 (17.9, 20.0)	6,923	107,437,176	23.7 (22.6, 24.8)
Race and ethnicity						
Non-Hispanic White	11,154	145,047,134	67.1 (66.8, 67.5)	22,841	318,130,603	69.7 (69.1, 70.3)
Non-Hispanic Black	2,827	24,670,920)	11.4 (11.3, 11.6)	3,924	35,777,637	7.8 (7.6, 8.1)
Non-Hispanic Other	1,345	16,726,674	7.7 (7.5, 7.9)	2,748	36,684,307	8.0 (7.6, 8.4)
Hispanic	2,877	29,569,802	13.3 (13.4, 14.0)	6,122	65,735,716	14.4 (13.9, 14.9)
Body mass index						
Underweight	440	4,205,602	1.9 (1.7, 2.2)	668	6,698,641	1.4 (1.2, 1.6)
Normal weight	5,907	67,815,856	31.2 (30.2, 32.2)	12,230	151,174,359	31.8 (30.7, 32.9)
Overweight	5,928	73,796,358	34.0 (33.0, 34.9)	12,516	165,595,994	34.9 (33.9, 35.8)
Obese	6,237	72,122,821	33.2 (32.2, 34.2)	12,366	151,628,290	31.9 (30.8, 33.0)
Self-perceived overall health						
Excellent, very good, good	16,177	198,099,451	88.5 (87.7, 89.3)	34,769	446,878,838	91.7 (91.2, 92.3)
Fair or poor	2,722	26,425,189	11.8 (11.1, 12.5)	3,809	40,268,418	8.3 (7.7, 8.8)
Self-perceived physical health						
Excellent, very good, good	15,698	192,615,730	86.0 (85.2, 86.8)	34,072	438,697,884	90.1 (89.4, 90.7)
Fair or poor	3,208	31,369,778)	14.0 (13.2, 14.8)	4,502	48,283,211	9.9 (9.3, 10.5)
Disease status						
Have a health condition	7,515	97,212,868	43.6 (42.5, 44.7)	12,982	190,555,990	49.5 (48.6, 50.4)
Do not have a health condition	11,298	125,668,494	56.4 (55.3, 57.5)	15,861	194,160,940	50.5 (40.5, 51.4)
Ever tobacco use						
Users	16,109	163,500,811	74.1 (72.9, 75.2)	32,376	349,767,806	72.9 (71.5, 74.2)
Non-Users	2,690	57,266,636	25.9 (24.8, 27.1)	5,977	130,270,464	27.1 (25.7, 28.5)
Established tobacco use						
Current users	4,548	29,157,918	15.7 (15.2, 16.3)	12,032	78,359,323	17.0 (16.5, 17.6)
Former users	3,761	52,422,407	28.3 (37.1, 29.5)	8,158	118,808,791	25.8 (24.6, 27.0)
Non-Users	6,232	103,682,140	56.0 (54.6, 57.4)	15,697	263,144,362	57.2 (55.7, 58.6)
Overall tobacco use						
Ever tobacco users	5,232	65,269,066	29.6 (28.7, 30.5)	12,186	152,599,692	31.8 (30.7, 32.9)
Current established users	7,080	45,521,326	20.7 (20.1, 21.2)	12,032	78,359,323	16.3 (15.8, 16.9)
Former established users	4,069	56,477,031	25.6 (24.6, 26.7)	8,158	118,808,791	24.7 (23.6, 25.9)
Never users	2,405	53,096,669	24.1 (22.9, 25.3)	5,977	130,270,464	27.1 (25.7, 28.5)
Exposure to secondhand smoke						
Exposed	11,438	105,400,159	47.4 (46.1, 48.7)	19,929	195,618,689	40.4 (39.4, 41.4)
Non-Exposed	7,287	117,010,632	52.6 (51.3, 53.9)	18,331	288,507,534	59.6 (58.6, 60.6)
Established e-cigarettes use						
Current users	1,146	7,177,916	3.3 (3.0, 3.6)	2,026	13,155,716	2.8 (2.6, 3.0)
Former users	864	5,140,523	2.4 (2.2, 2.6)	3,028	18,152,270	4.0 (3.7, 4.2)
Non-e-cigarettes users	15,407	202,355,999	94.3 (93.9, 94.6)	29,501	428,171,806	93.2 (92.8, 93.6)
Regular e-cigarettes flavor exclusively used among current users						
Menthol or mint	183	1,120,991	0.5 (0.4, 0.6)	247	1,716,503	0.4 (0.3, 0.5)
Tobacco ^c^	-	-	-	311	2,174,706	0.5 (0.4, 0.6)
Fruit	195	1,127,848	0.5 (0.5, 0.6)	576	3,652,209	0.8 (0.7, 0.9)
Candy or sweet	89	559,428	0.3 (0.2, 0.3)	328	2,052,456	0.5 (0.4, 0.5)
Other (clove or spice, chocolate, alcoholic drinks, other)	73	482,460	0.2 (0.2, 0.3)	83	527,569	0.1 (0.1, 0.2)
Multiple flavors	271	1,661,951	0.8 (0.7, 0.9)	471	2,966,375	0.7 (0.6, 0.8)
Regular e-cigarettes flavor exclusively used among former users						
Menthol or mint	90	512,890	0.2 (0.2, 0.3)	197	1,215,763	0.3 (0.2, 0.3)
Tobacco ^c^	-	-	-	205	1,417,855	0.3 (0.3, 0.4)
Fruit	54	330,659	0.1 (0.1, 0.2)	306	1,719,018	0.4 (0.3, 0.4)
Candy or sweet	25	173,810	0.1 (0.04, 0.1)	132	724,750	0.2 (0.1, 0.2)
Other (clove or spice, chocolate, alcoholic drinks, other)	15	85,609	0.04 (0.02, 0.06)	74	446,462	0.1 (0.07, 0.1)
Multiple flavors	82	491,126	0.2 (0.2, 0.3)	284	1,681,153	0.4 (0.3, 0.4)
Marijuana use						
Users	1,750	13,889,248	6.6 (6.0, 7.3)	4,160	35,555,954	11.5 (11.0, 11.9)
Non-Users	14,608	194,901,724	93.3 (92.7, 94.0)	33,780	431,818,610	88.5 (88.0, 89.0)

^a^ Missing data. Total frequencies do not add to 18,925 as all variables show missing data. The rate for missing data for individual participants ranges between 0.01% (age) to 23.2% (established tobacco use).

^b^ Missing data. Total frequencies do not add to 38,638 observations as all variables show missing data. The rate for missing data for the total observations used in the analysis ranges between 0.09% (sex) to 25.3% (disease status).

^c^ The tobacco flavor category was not assessed at baseline in the PATH Study. Counts in the tobacco flavor category for total observations in the study include participants reporting ECIG tobacco flavor use during the PATH Study Waves 3–5.

### Data analyses

First, data analyses included weighted descriptive statistics to report baseline characteristics of the study population and the weighted incidence proportion (WIP) of nocturnal dry cough. Complex survey all-waves weights and 100 replicate weights from Wave 5 were used to generate weighted representative estimates of the US population. The balanced repeated replication method was used to construct replicate weights with Fay’s adjustment of 0.3 to improve the stability of estimates as recommended by the PATH Study. One hundred replicates were generated to obtain weighted percentages for all sample baseline characteristics and WIP of nocturnal dry cough. Second, weighted Rao-Scott chi-squared tests were conducted to examine associations among covariates by ECIG flavor exposure and nocturnal dry cough. Third, generalized estimating equations (GEE) models were used to quantify unadjusted and adjusted associations between ECIGs flavors use and dry cough P12M accounting for time-varying variables. Here, weighted percentages, unadjusted, and adjusted relative risks and their 95% confidence intervals (95% CIs) are reported. Our models used three clustered measurements of each follow-up period and from each subject. In this analysis, all variables represent time-varying measurements, with different values at each follow-up period. Rates of data missingness are low in The PATH Study. Most of the missing data in this analysis are due to variables being recoded to missing data when participant responses were “do not know” or “refused to answer”. Missing rates are reported. The models provided weighted estimates of relative risks for complete cases only. GEE provided estimates of associations from a single sample analysis, while allowing to control for interdependence among individual observations. Two-side p-values of <0.05 were considered statistically significant. Analyses were conducted using SAS v9.4 (SAS Institute Inc., Cary, NC).

## Results

### Sample description

Weighted estimates of the baseline sociodemographic and health characteristics of participants at Wave 2 are shown in Table 1. Briefly, 35.3% of the population were young adults, 34.6% were middle aged adults, 52.2% were females, 37.7% achieved high school level or less, 67.1% were non-Hispanic White, and 43.6% reported a health condition. In terms of overall tobacco use, 24.1% were never tobacco users; 20.7% and 25.6% were current and former established tobacco users, respectively. Among participants, 2.9% were classified as current established ECIGs users, 4% as former established ECIGs users, and 92.8%, as non-ECIGs users.

### Incidence of nocturnal dry cough

Except for age and health insurance coverage, all potential confounders were associated with self-reported nocturnal dry cough as observed in Table 2. Among never tobacco users, the WIP was 10.2% (95%CI 9.1, 11.3); current and former established tobacco users showed WIP of 15.1% (95%CI 13.3, 16.9) and 11.9% (95%CI 10.0, 13.8), respectively. Among established ECIGs users, current users showed a higher WIP than former users, 14.8% (95%CI 13.1, 16.6) in contrast to 8.9% (95%C 8.2, 9.7). Table 3 shows the WIP of nocturnal dry cough by ECIGs flavors category. Compared to non-ECIGs users (WIP:11.1%; 95%CI 10.6, 11.6), the WIP of nocturnal dry cough was significantly higher among current (WIP:16.6%; 95%CI 10.5, 21.2) and former fruit flavored ECIGs users (WIP:16.6%; 95%CI 11.3, 21.9). Across all flavor categories in former ECIGs users, the WIP of cough was higher than the non-ECIGs counterpart.

**Table 2:** Weighted Incidence Proportion (WIP) of nocturnal dry cough among current and former established e-cigarettes users by potential confounders, PATH study, Waves 1–5 (2013–2019), n = 38,638 observations.

Variables	Cases ^a^	Weighted Frequency	WIP ^b^	95% CI	*p* value ^c^
Age					
18–24	739	4,278,964	11.6	10.6, 12.6	0.0886
25–34	1,240	10,427,170	11.4	10.5, 12.3	
35–44	804	8,580,680	10.1	9.2, 11.1	
45–54	778	10,181,079	11.9	10.9, 13.0	
55–64	745	10,636,154	12.1	10.9, 13.4	
≥65	551	11,902,119	11.7	10.6, 12.9	
Sex					
Male	2,213	25,924,640	10.9	10.3, 11.5	0.037
Female	2,642	30,058,082	12.0	11.3, 12.7	
Highest educational level achieved					
High school or less	1,858	21,347,607	12.8	12.0, 13.6	<.0001
Some college or associate degrees	1,769	17,902,590	11.8	11.1, 12.6	
Bachelor’s degrees or higher	1,220	16,636,297	9.9	9.2, 10.5	
Currently covered by health insurance					
Yes	4,157	49,936,674	10.3	9.9, 10.7	0.8338
No	671	5,805,461	1.2	1.0, 1.4	
Total household income in the past 12 months (US$)					
<10,000	722	5,944,682	15.1	13.7, 16.6	<.0001
10,000–24,999	1,042	10,549,445	14.2	12.8, 15.5	
25,000–49,999	1,092	12,617,098	12.7	11.7, 13.8	
50,000–99,999	1,094	14,062,778	10.6	9.7, 11.5	
>100,000	677	9,677,918	9.0	8.2, 9.9	
Race and ethnicity					
Non-Hispanic White	2,858	36,138,629	11.4	10.8, 11.9	<.0001
Non-Hispanic Black	606	5,395,897	15.1	13.3, 16.9	
Non-Hispanic Other	366	4,356,935	11.9	10.0, 13.8	
Hispanic	681	6,694,403	10.2	9.1, 11.3	
Body mass index					
Underweight	89	852,450	12.7	9.2, 16.2	<.0001
Normal weight	1,411	15,129,707	10.0	9.2, 10.8	
Overweight	1,513	18,939,273	11.4	10.7, 12.2	
Obese	1,743	19,888,592	13.1	12.1, 14.1	
Self-perceived overall health					
Excellent, very good, good	3,974	47,058,705	10.5	10.1, 11.0	<.0001
Fair or poor	878	8,900,640	22.1	20.5, 23.7	
Self-perceived physical health					
Excellent, very good, good	3,894	46,133,412	10.5	10.1, 11.0	<.0001
Fair or poor	959	9.842,152	20.4	18.9, 21.9	
Disease status					
Have a health condition	2,345	30,112,748	15.8	15.0, 16.7	<.0001
Do not have a health condition	1,675	17,892,967	9.2	8.6, 9.8	
Ever tobacco use					
Users	4,274	43,169,258	12.3	11.9, 12.8	<.0001
Non-users	557	12,060,576	9.2	8.2, 10.3	
Established tobacco use					
Current users	2,197	13,764,785	16.4	15.6, 17.1	<.0001
Former users					
Non-users	2,655	42,192,295	10.5	9.9, 11.0	
Overall tobacco use					
Ever tobacco users	2,858	36,138,629	11.4	10.8, 11.9	<.0001
Current established users	606	5,395,897	15.1	13.3, 16.9	
Former established users	366	4,356,935	11.9	10.0, 13.8	
Never users	681	6,694,403	10.2	9.1, 11.3	
Exposure to secondhand smoke					
Exposed	3,034	28,439,902	14.5	13.8, 15.2	<.0001
Non-Exposed	1,768	27,199,030	9.4	8.9, 10.1	
Established e-cigarettes use					
Current users	307	1,950,901	14.8	13.1, 16.6	<.0001
Former users	1,298	13,454,639	8.9	8.2, 9.7	
Non-e-cigarettes users	2,799	37,588,922	12.4	11.7, 13.1	
Marijuana use					
Users	645	5,210,394	1.1	1.0, 1.3	<.0001
Non-users	3,442	46,403,906	10.0	9.6, 10.5	

^a^ Cases. Frequencies represent the total number of observations reporting nocturnal dry cough among each variable category.

^b^ WIP. The weighted incidence proportion is calculated dividing the number of cases of nocturnal dry cough by the number of observations at risk over the follow-up period). The denominators for each calculation are the total observations for each variable category shown in Table 1.

^c^ *p* values correspond to Rao-Scott chi-squared tests.

**Table 3:** Weighted Incidence Proportion (WIP) of nocturnal dry cough among current and former established e-cigarettes users by flavors regular or last used, PATH study, Waves 1–5 (2013–2019), n = 38,638 observations.

Type of e-cigarettes User (n)	Type of Regular Flavor Used (n) ^a^	Nocturnal Dry Cough in the Past 12 Months		
		Frequency	WIP	(95% CI)
Non- e-cigarettes Users	No applicable (28,629)	3,395	11.1	10.6, 11.6
Current Established (1,930)	Menthol and mint (247)	43	15.8	10.5, 21.2
	Tobacco (265)	46	14.1	8.5, 19.7
	Fruit (576)	97	16.6	13.3, 19.9
	Candy and sweet (288)	40	13.2	8.9, 17.4
	Others (83)	9	10.0	2.4, 17.5
	Multiple (471)	71	14.5	10.9, 18.1
Former Established (1,198)	Menthol and mint (197)	36	15.3	10.5, 20.2
	Tobacco (205)	38	17.0	11.8, 22.3
	Fruit (306)	53	16.6	11.3, 21.9
	Candy and sweet (132)	22	19.5	13.1, 25.9
	Others (74)	18	27.1	13.6, 40.6
	Multiple (284)	59	22.6	17.7, 27.4

Rao-Scott Chi-Square = 85.07, 12 DF, *p*≤0.0001

^a^ The final analytical sample in this table was 31,843. The sample size for each category includes observations with complete data for type of regular tobacco used and reporting of dry cough. Sample sizes in parenthesis indicate the denominator used to calculate the weighted incidence proportion.

### Longitudinal associations by ECIGs flavor use

In Table 4, after controlling for all sociodemographic variables, health status, marijuana and overall tobacco use, current ECIGs users of fruit flavors showed 40% higher risk of reporting nocturnal dry cough than non-ECIGs users (aRR:1.40, 95%CI 1.01, 1.94). Current menthol and mint ECIGs users showed adjusted relative risk of 1.26 (95%CI 0.77, 2.07) compared to non-ECIGs users. Former ECIGs users of multiple flavors and other flavors had 233% and 66% higher risk to develop cough, respectively (aRR:3.33, 95%CI 1.51, 7.34 and aRR:1.66, 95%CI 1.0.9, 2.51), relative to non-ECIGs users.

**Table 4:** Association between type of regular flavor used and incident cases of self-reported nocturnal dry cough in the past 12 months, PATH study, Waves 1–5 (2013–2019).

Type of ECIGs User (n)	Type of Regular Flavor Used (n)	Unadjusted		Adjusted	
		RR	95%CI	RR	95%CI
Non-ECIGs Users	No applicable (28,629)	Reference		Reference	
Current Established (1,930)	Menthol or mint (247)	1.51	1.00, 2.28	1.26	0.77, 2.07
	Tobacco (265)	1.32	0.87, 2.00	0.87	0.55, 1.37
	Fruit (576)	1.60	1.24, 2.06	1.40	1.01, 1.94
	Candy and sweet (288)	1.22	0.83, 1.79	0.87	0.53, 1.43
	Multiple (471)	0.89	0.41, 1.90	0.50	0.19, 1.29
	Others (83)	1.36	1.01, 1.83	0.98	0.67, 1.44
Former Established (1,198)	Menthol or mint (197)	1.45	0.97, 2.18	1.08	0.63, 1.84
	Tobacco (205)	1.65	1.11, 2.44	1.38	0.88, 2.17
	Fruit (306)	1.60	1.12, 2.26	1.50	0.94, 2.40
	Candy and sweet (132)	1.95	1.19, 3.18	1.50	0.77, 2.92
	Multiple (284)	2.98	1.61, 5.53	3.33	1.51, 7.34
	Others (74)	2.34	1.67, 3.26	1.66	1.09, 2.51

## Discussion

Our findings underscore the contribution of fruit flavors in ECIGs to the development of nocturnal dry cough among current established ECIGs users, after controlling for sociodemographic variables (sex, age, race-ethnicity, educational level, household income), disease status (heart disease, pulmonary disease, gum disease, cancer, precancerous oral lesions, ulcer, gastrointestinal bleeding, cataract and glaucoma, or diabetes mellitus), marijuana use, overall tobacco use, and secondhand smoke exposure. Our results are consistent with previous research from our group showing that ECIGs users of fruit were more likely to report dry cough in the P12M, as compared to non-established ECIGs users. Initially, concerns regarding ECIGs exposure were focused on the effects of nicotine, as nicotine-containing ECIGs show a dual effect on cough: initial coughing and a delayed suppressive effect on coughing [40]. However, exposure to flavors and flavorings from ECIGs are of special concern for cough.

Flavors in ECIGs evoke chemosensory experiences that has been associated with initiation, progression and dependence or addiction to ECIGs products among youth, young adults, and never smokers [47, 48]. Flavors enhance the palatability of high concentrations of ECIGs constituents, such as nicotine, PG and VG [15, 49, 50]. Characterizing flavors are vastly available in the market and are grouped into 11 main categories, including the most popular “fruit”, “dessert-candy-sweets”, “tobacco”, and “mint/menthol” [51]. Fruit flavors are more likely to attract youth and young adults [52, 53]. Flavorings, the chemicals responsible for the perception of flavors, are derived from the food industry and generally recognized as safe for ingestion, but not inhalation [54]. However, some flavorings including benzaldehyde (cinnamon), cinnamaldehyde (cinnamon), ethyl methylphenylglycidate (fruit [strawberry]) diacetyl, ortho-vanillin (sweet), ethyl vanillin (sweet), and dl-menthol have shown to elicit oxidative stress and pro-inflammatory effects in the respiratory system when inhaled [15]. Evidence from *in vitro* and animal studies have shown that flavorings can elicit cytotoxic effects and abnormal activation for the airways epithelial cells, increased production of antimicrobial molecules, impaired mucociliary clearance, and damped immune responses [15]. These effects provide biological plausibility to support the association between ECIGs use and coughing. ECIGs flavorings can dysregulate the respiratory innate immunity and elicit inflammation, which are associated with coughing.

Cough, a component of the airway innate immunity, is a neuro-physiological response to noxious stimuli initiated by ion channels, mainly transient receptor potential ankyrin 1 (TRPA1) and transient receptor potential vanilloid 1 (TRPV1) [55]. As our study observed the highest relative risk of nocturnal cough among current ECIG users vaping fruit flavors, we have to consider the ingredients of fruit flavors. Two popular flavorings present in fruit flavors, cinnamaldehyde and menthol, might disrupt the airway’s epithelia, elicit oxidative stress, impair mucociliary clearance, and induce inflammatory responses by increasing the release of inflammatory cytokines and through the activation of TRPA1 [15, 56, 57]. This would be potentially augmented by the inclusion of synthetic cooling/ice agents, such as WS-3 (N-Ethyl-2-isopropyl-5-methylcyclohexanecarboxamide), WS-23 (ethyl diisopropyl propionamide), and coolada, present in US-marketed fruit flavored refill e-liquids [58]. WS-3 and WS-23 have been shown to cause more oxidative and inflammatory responses in lung epithelial cells [58–60]. The presence of additives/adjuncts in the flavored products is an important consideration when evaluating the potential health effects from ECIGs.

Coughing is an unpleasant, disturbing, and distressing symptom, which is difficult to manage and requires considerable costs and health care utilization [61]. Further, coughing is the most common reason for medical visits in primary care [61]. Nocturnal cough is a common symptom of respiratory and non-respiratory disease with multiple etiologies, including asthma, allergies, and environmental exposures such as tobacco smoke and secondhand smoke. Though not all cough is associated with later adverse respiratory outcomes, cough is suspected to be “the canary in the coal mine” with regards to the toxicity of ECIGs in the respiratory system [62]. The “vaping cough”, as referred by the general public, is one of the most common symptoms reported by ECIGs users. The “vaping cough” is thought to be related to the throat hit, i.e., a harsh and irritating sensation in the throat, [63] caused by higher concentrations of nicotine in newer ECIG devices. However, symptoms like cough or dry throat were not different among ECIG users reporting strong throat hit as compared to those with weak throat hit [64]. In addition, ECIG users reporting cough as a frequent symptom associated to their ECIG use was related to high levels of VG in the e-liquids and the presence of the flavor cinnamon [65]. King and colleagues showed that among ECIGs users, 40% of the adults and 42.3% of the adolescents reported cough as the most frequent symptom attributed to ECIGs use [66]. Our report is one of the first studies examining the potential contribution of flavors in the production of cough.

Our population-level study focused on the association between ECIGs and cough, as an early marker of respiratory health. Our study used GEE models, a statistical method to provide estimates of a relative risk assessment in a prospective longitudinal analysis of a national representative sample of the US population. Though we have adjusted for the most relevant potential confounders described in the literature, including sociodemographic characteristics, health status, tobacco use, marijuana use, and second-hand smoke exposure, there is still potential for residual confounding from other variables. To account for confounding from tobacco use, our statistical models qualitatively assessed overall tobacco use and ECIGs flavor in all analyses. To minimize the impact of current or former tobacco use on studies of ECIGs, we recommend that future studies include quantitative estimations for tobacco use, e.g., frequency of smoking or cigarette packs per year. In addition, race and ethnicity specific statistical models were not conducted as sample sizes were too small for comparisons across ECIG flavor categories; however, future studies need to consider exploring the impact of these structural factors, particularly in light of the study by Connolly et al. which observed that Spanish-speaking participants were significantly less likely to be asked by providers about e-cigarette use compared to English speaking patients [67].

The toxicological profile for most of the flavors remains difficult to define [8, 68]. Our study did not perform a direct comparison among the different broad flavor categories assessed in the PATH Study. The 95% confidence intervals for the adjusted relative risk shown in the study suggest there is no statistical difference among the categories examined in the study. However, future research might address specific comparisons between flavor categories, considering potential confounders such as sociodemographic variables, behaviors associated with ECIG use, tobacco use history, secondhand smoke exposure, and marijuana use. The expanding evidence suggest that menthol, strawberry, cinnamon, and tobacco flavors might elicit biological changes associated with respiratory adverse effects [12]. There is emerging evidence for increased risk of respiratory disease in ECIGs users, though the observed risk is generally lower than that for cigarettes. However, the relatively short time that ECIGs have been on the market compared to cigarettes means that, even when controlling for length of use, the sample of ECIGs users with sufficiently long exposures to lead to respiratory disease will be smaller in comparison to their age-matched peers who smoke cigarettes. This discrepancy limits the ability to compare the relative risks between smoking and ECIGs. Thus, there is a need to characterize more proximal markers of later disease development, such as cough and other physiologically relevant respiratory symptoms. As the availability and types of flavors evolves in response to regulation, future studies will need to continue focusing on flavor-specific effects on respiratory health, including early indicators such as cough.
